# Elementa Medicinæ Practicæ

**Published:** 1836-01

**Authors:** 


### BRITISH AND FOREIGN

### MEDICAL REVIEW,

FOR JANUARY, 1836.

### PART FIRST.

### Analytical and Critical Reviews.

### Art. I.

Francisci Bene, Med. Doct., Consiliarii Regii, Professoris P. O. Therapiæ Specialis ac Praxis Medicæ, et Senioris Facultatis Medicæ in Regia Scientiarum Universitate Hungarica, Elementa Medicinæ Practicæ. *E Prælectionibus illius publicis Edita per* Franciscum Bene, Jun. m.d.T$^{i}$. 5.—8vo. *Pestini*, 1833, 1834. Adolphi Hartleben.

*Elements of Practical Medicine, by* Franciscus Bene, m.d. &c. &c., Professor in the Royal Hungarian University of Sciences, &c. *Edited from his public Lectures, by* Franciscus Bene, Jun. m.d. Five vols.—8vo.

This work is the substance of Lectures on the Practice of Medicine, delivered by the Professor in the University at Pest, who, as we are informed in the preface, has held his office seventeen years, and given systematic and clinical lectures regularly, but has been too much occupied in practice to undertake their publication. They are published from the notes taken by his son; and, we may presume, at least in their general outlines, submitted to his correction. We have examined it with some care, as an interesting evidence of the state of the science and practice of medicine in the Austrian dominions; a part of Europe which is generally regarded as one of the least enlightened, but in which every encouragement is given to the acquisition of scientific knowledge, and in which the plan of medical education, in particular, is regarded by some as a model for the imitation of other countries.

We must confess that we have been considerably disappointed in examining the contents of the work, as an indication of the general principles on which the science is taught; and, after we have given some account of the work, we think our readers will agree with us in opinion, that, although quite sufficiently indicative of learning, industry, and much useful professional knowledge, and of a conscientious desire to communicate useful instruction, it is yet considerably deficient in the simplicity and precision, and even, in some points, in the specific information, which are desirable in such a publication at the present day.

That such deficiencies should be found in the medical instruction of a country where, as we have understood, scientific education is made an object of primary importance by the government, is a fact to which we think it of some consequence, in these days of projected reforms in our medical schools and institutions, to direct the attention of our readers: not because we doubt that great benefit may result from judicious reforms, nor because we dissent from the opinion, (now, we believe, pretty general in the profession,) that the regulation of such matters deserves more attention than it has yet received from the government of this country; but because we think it important that all who take an interest in the subject should be aware, that, by merely increasing the amount of learning, and by varied scientific acquirements, included in the compulsory education of medical practitioners, we cannot by any means ensure increased accuracy, or extent of information, or sounder judgment, in the apprehension or application of those medical facts and principles with which the interest of the public demands that they should be most intimately acquainted. The proper education of a medical man requires, in fact, the exercise not merely of those faculties by which facts are observed and knowledge is acquired, but especially of those by which these facts are weighed and compared, a right judgment formed of their comparative value and importance, and the right inferences deduced from them. We believe it to be certain that, in some schools of medicine, the former class of the human faculties have been cultivated to a certain degree at the expense of the latter; otherwise, we do not see how it could have happened that the schools of France, Germany, and Italy, in which, certainly, a much greater amount of scientific knowledge, connected with medicine, has been required of the students, than most British licentiates, or even graduates, possess, should nevertheless have failed in producing a body of practitioners more deserving, or equally deserving, of the confidence of their fellow-citizens, in the ordinary exercise of their profession.

This work commences by a preliminary dissertation (Prolegomena), consisting of two parts: the first, a brief history of medical literature, from the earliest times; the second, a manual of general instructions for the examination of patients, and the other duties of a practitioner.

The first of these sections seems to be hardly more than an index to Lectures delivered on the subject, with lists of the authors regarded as most characteristic of the different eras in the progress of medicine, but little attempt to discriminate their respective contributions to the science. On this part of the work we shall only remark, as evidence of the defective information in regard to British medical writings, that, although his notice of French systematic authors comes down to Andral and Cruveilhier, and that of English to Mason Good and Mackintosh, although he is somewhat particular as to periodical medical literature, and notices various British works on special subjects, yet we find no mention of Young, of Parry, of Wilson Philip, of Armstrong, of Brodie, Travers, Marshall Hall, or Bright, in London; none of Hamilton, Thomson, Abercrombie, or Alison, in Edinburgh; of the Burns's, in Glasgow; or of any one of the physicians or surgeons of Dublin: none of the Transactions of the College of Physicians, the Medico-Chirurgical Society in London, nor of the Dublin Transactions or Hospital Reports; and, although due notice is taken of the Lancet and Medical Gazette, Dr. Johnson's Medico-Chirurgical Review is passed over *sub silentio*. It is true that many of these authors are named as authorities on special subjects, in the subsequent parts of the work; but several of them, at least, would surely have appeared, to one well acquainted with their writings, equally entitled to notice in a general review of the progress of the science, as Brown, Beddoes, or Trotters, or even as Abernethy, Wardrop, or Lawrence, whose names are here introduced.

The second part of this preliminary dissertation, termed "Occupatio Medici ad lectos Ægrorum," is perhaps of more value: it contains general rules for the guidance of young practitioners; then a full enumeration of the chief symptoms to be looked for, as indicating derangement, first in the function of digestion, and all the functions of parts contained in the abdomen and pelvis; next in the thoracic viscera and the circulation; and, lastly, in the organs of sense and the nervous system. Then comes a general statement of the causes of disease, predisponent and exciting, hereditary constitution, age, sex, temperament, climate and locality, diet, habits, occupations, atmospheric changes, contagions, diarrhœa, poisons, injuries, mental exertions, &c.; all which demand attention, even on the first examination of patients;—and then general advice as to the manner of combining and reflecting on the information thus obtained, so as to form a judgment of the prognosis and of the treatment suited to the case.

All these are subjects to which it is of real importance to direct the attention of students, even in the outset of a course of practical instruction; but it is equally obvious that they must be as yet unprepared either to understand or profit by any minute detail of symptoms, and, therefore, that a prudent discrimination is very necessary in placing these topics before them; the enumeration of particulars should not be too ample and minute, and pains should be taken to classify the symptoms enumerated, and distinguish those which are of prominent and essential importance from those which are of a secondary or accidental character. This is hardly attempted in the present instance: even the broad line of distinction drawn by several previous authors, in such a general enumeration of symptoms, between those which are taken from deranged functions or altered external appearances, of which the practitioner can judge for himself,—and those which consist in uneasy sensations, of which he must be informed by the patient, (although, in subsequent parts of the work, e. g. in t. v. § 12, the author shows his sense of its value,) is here neglected; and it is hardly necessary to say, that it is one easily explained, even to young students, and of great importance to young practitioners, particularly in the investigation of nervous, hysterical, or hypochondriacal complaints.

Besides, in this section we observe several omissions, of such a nature as to shew that the author could not have been aware of the real importance of the symptoms in question. For example: although there is a whole paragraph (§ 33,) on symptoms in the condition of the urine, we find no mention made either of the means of detecting albumen in it, or of the importance of taking its specific gravity. Although percussion on the abdomen is mentioned, (§ 36,) it seems to be merely with the view of distinguishing fluid effusion from tympanitis: there is no hint of the importance of the dull sound on percussion, as indicating solid enlargements of viscera. The immediate objects of percussion of the thorax and auscultation are very imperfectly stated in § 39; and, although no less than twenty-five different characters are enumerated in § 38, as demanding attention in the examination of the pulse, there is no mention of the simple and frequently decisive observations which may be made, either on the exact position of the apex of the heart, or on the nature of the pulsation in the subclavian arteries.

The general arrangement of the subjects treated in each of the separate chapters is distinct and uniform; and, although the continual repetition of the indications of cure,—first, the removal of exciting causes; then the correction of the "character dynamico-organicus" of the disease, whether sthenic or asthenic, &c.—is somewhat tiresome; yet the necessity of attention to points of real practical importance, and of varying the treatment according to the condition of the patient, as well as the name and nosological position of the disease, is always kept steadily in view. The authors, on whom reliance is chiefly placed for the facts stated in each chapter, are regularly enumerated.

After the preliminary dissertation, the author proceeds to lay down his "Doctrina de Febribus," which he arranges in the following manner. First, we have a chapter entitled "Febris in genere," containing a description of the characters of the febrile paroxysm, whether idiopathic or symptomatic, and of its external causes, (where, by the way, we have exposure to heat or cold, to malaria or contagion, all put together under one head, and *seven* other general causes enumerated, we apprehend, very needlessly); then we have an outline of the grounds of distinction among fevers, as idiopathic or symptomatic, remitting or continued, sthenic or asthenic, simple or complex; of the modes of their terminations, of the general signs of these terminations, guiding the prognosis, and even of the general indications of treatment, and especially of the antiphlogistic regimen, and the modifications it requires in the progress of febrile disease.

We have then, in three distinct sections, a full discussion of the history and treatment of what are called the *first* order of fevers, "Febres Continuæ Cardinales," viz. "cum charactere inflammatorio," "septico," and "nervoso." In the two latter we have a very accurate description of the usual forms and chief varieties of typhoid fever; and we would notice, in particular, the distinction drawn in the last section between the "Febris nervosa cum erethismo" and the "Febris nervosa cum torpore," as a decided improvement on the French mode of classing these varieties together, under the head of "Fièvre Ataxique."

But, after a full and formal discussion of these cardinal fevers, we are next informed that these are of very rare occurrence, unless when combined with other morbid conditions; and then we are immediately presented with histories and descriptions, drawn up in the same formal style, of the Febris gastrica, of which four distinct forms are given; the saburralis, the biliosa, the mucosa, and verminosa; then of the Febris catarrhalis and Febris rheumatica. These constitute his *second* order, the "Febres Continuæ Compositæ;" and then we have the *third*, the "Febres Continuæ Contagiosæ," comprising the Typhus Contagiosus Europæus, the Pestis Orientalis, and the Febris Flara Americana;—and, after all these, we have the *fourth* order of fevers, the "Febris Intermittens;" comprehending Remittent Fevers, such as are described by Torti.

It is to be observed, that under several of these heads, (particularly the Febris gastrica biliosa, § 116, and mucosa, § 123, catarrhalis, § 135, and rheumatica, § 150,) fevers are enumerated which take the distinctly typhoid type; but it will be easily understood that the cases, under each of these heads, which have the inflammatory form of fever, are not to be distinguished from what we usually call inflammatory diseases,—the Phlegmasiæ and Profluvia of Cullen,—Hepatitis, Enteritis, (especially the Enteritis erythematica of that author,) Catarrh, or Bronchitis, Dysentery, and acute Rheumatism.

On the theory of fever, Professor Bene is laudably brief; but he expresses, we think, too decidedly his opinion, that its external causes act primarily on the nervous system, and only through its intervention excite changes in the vascular system and in the blood. These changes in the state of the circulation, though often excessive and injurious, he thinks an essential part of the process, by which the original deleterious impression on the nervous system is removed; and, conformably with these principles, he regards critical evacuations as the sign, not the cause, of the abatement of fevers. (See § 71.)

The author is at much pains to state the variety of symptoms, and to explain the different alleged or probable causes of these varied forms of febrile disease; and it is right to add, that the practice he recommends comprehends what we regard as the most important remedies, and is judiciously varied according to the type and period of the disease in different cases. We observe much use made, in the earlier stages of almost all cases, of the tartar emetic. Calomel is used sparingly and cautiously: it is recommended in the Febris *mucosa,* but not in the *biliosa* nor in the *saburralis;* and for this difference we see no reason assigned. Purgatives in general are recommended less freely than with us, but not less freely than by other judicious practitioners in the southern parts of Europe. Less stress is laid on wine, and more on stimulating medicines, (particularly the infusion of the root, and decoction of the flowers, of the Arnica,) than we believe to be judicious. We have no doubt that he is too timid as to the use of opium in the advanced stages of the Febris gastrica mucosa, comprehending, as it must do, many cases of what we call severe diarrhœa and dysentery. His directions in regard to bloodletting would likewise lead, we are persuaded, to inefficient practice in the strictly inflammatory cases; but they are generally given in terms necessarily indefinite, so that, without actually seeing his practice, it is difficult to form a judgment of them.

The greatest and most fundamental deficiency is in the department of morbid anatomy, and in the general views of the kind of danger to be apprehended in these various cases, which can only be deduced from a knowledge of that part of the history of these diseases. The morbid appearances to be expected after each of the species considered are indeed enumerated, but in a vague and indefinite manner; and little attempt is made to connect peculiar appearances with peculiar symptoms, so as to give precision to our ideas of the danger threatening in individual cases. For example, under the head of Febris catarrhalis (including all forms of idiopathic and symptomatic bronchitis,) it is indeed quite correctly stated, in general terms, that the danger depends partly on the extent of inflammation and effusion in the trachea and bronchi, and partly, in many cases, on the "nervous or septic (i. e. typhoid) characters of the fever." (§ 137.) But of the means of judging of the extent to which the bronchial inflammation, and its results, may have gone, and even of the distinction of that inflammation from peripneumony, of the special characters of bronchitis in the dead body, and its frequent combination with œdema or with emphysema of the lungs, or with that which takes place in the very last stage of life, ("peripneumonie des agonisans;") and of the importance of these principles in giving precision to our prognosis and practice in many individual cases, nothing is said. Again, as to the connexion of rheumatism with internal inflammations, we find this statement:

"Ubi febris rheumatica characterem inflammatorium eminentiorem habet, ibi frequentissime adest inflammatio rheumatica membranarum serosarum aut fibrosarum in cavis internis corporis, atque partim quod secretio morbosa cito contingit, partim quod status inflammatorius visceribus ad vitam necessariis communicatur, exponitur æger summo vitæ periculo; quod etiam locum habet dum morbus mitior rheumatismum articularem externum constituens subito disparet, atque status morbosus in meningibus, imprimis in dura matre, aut arachnoidea, pleura, peritoneo, pericardio evolvitur."* (§ 152.)

There is no warning given, either of the very peculiar (almost exclusive) tendency of this inflammation to affect the heart or pericardium, or of the indications, frequently so obscure, of the commencement of that affection; neither is there, subsequently, any distinct statement of what we believe to be the general result of experience in such cases, viz. of the very frequent failure of general bloodletting, after the affection of the heart has commenced, and the greater efficacy of repeated local bleedings.

In regard to the morbid appearances in the Febris nervosa, we are told,

"Frequenter in tubo alimentari apparent signa gastro-enteritidis prægressæ, imprimis in intestino ileo inflammatio; sæpe conspiciuntur in tubo intestinali pustulæ inflammatæ, exulceratæ, crustis obtectæ, variolis similes; propterea vocatur ileitis pustulosa, ab aliis dothinenteritis. Sæpe tamen nec inflammatio, nec degenerationes glandularum tubi intestinalis reperiuntur, sed inflammationes aliarum partium, frequenter cerebri aut medullæ spinalis, quorum meninges exhibent vasa sanguifera valde turgida, sæpe serum vel sanguis vel *materia puriformis* in superficie cerebri vel ventriculis ejusdem vel in specu vertebrarum effusa sunt, subinde substantia tota cerebri mollior, aut durior reddita est. *Ubi tales desorganisationes non contingunt æger morti eripitur*, sed debilitatem experitur chronicam."† (§ 98.)

And nearly the same statement is repeated as to the morbid appearances in the Typhus contagiosus Europæus, with the addi-

* "When rheumatic fever has a more marked inflammatory character, there is most frequently rheumatic inflammation of the serous or fibrous membranes of the internal cavities of the body; and partly because morbid secretion soon takes place, partly because the inflammatory condition is communicated to viscera essential to life, the patient's life is exposed to the greatest danger; which is also the case when a milder rheumatic affection of the joints suddenly disappears, and the morbid condition is evolved in the meninges, particularly in the dura mater and arachnoid, in the pleura, the peritoneum, and the pericardium."

† "Signs of preceding gastro-enteritis often appear in the alimentary tube, and particularly of inflammation of the ileum; pustules are often observed, inflamed, ulcerated, covered with crusts, resembling variolæ, whence it is called *ileitis pustulosa*, and by others *dothinenteritis*. Often, however, neither inflammation nor degenerations of the intestinal glands are found, but inflammations of other parts, frequently of the brain and spinal marrow, of which the membranes exhibit highly turgid blood-vessels, and serum, or blood, or puriform matter is often effused on the cerebral surface, or in the ventricles, or in the cavity of the spine; the whole substance of the brain is occasionally softer or harder than natural. When such lesions do not take place, the patient recovers, but experiences long-continued debility."

tion that "in partibus internis deteguntur varia quæ indicant inflammationem prægressam in cavo faucium, trachea, bronchiis, pulmonibus, ventriculo, tubo intestinali, vel aliis visceribus cavi abdominis;"* (§ 163;) several of which statements, we are confident, could not have been made in this unqualified manner if the author had been at pains, first, to distinguish strictly morbid from pseudo-morbid appearances; and, secondly, to distinguish the symptoms of what he calls nervous fever and contagious typhus from those of encephalitis and other inflammatory diseases, treated in another volume.

We must observe, to the credit of our author, that he seems to have carefully studied the grand external causes of these febrile diseases, and the circumstances in which they chiefly operate, and gives judicious directions as to prophylaxis. His general conclusion as to yellow fever, that it is generally endemic, and produced by a poison generated on the earth's surface, and occasionally in ships, by the heat of the tropical sun, and then diffused through the atmosphere; but that, in certain circumstances, it becomes also contagious, and spreads, within certain limits, like the typhus of Europe; is, we apprehend, the safest and most probable which can be drawn from the facts hitherto known.

Having thus disposed of the first great class of diseases, viz. fevers, the author proceeds to treat of his second great class, Inflammations, first in general terms, and then as occurring in the head and organs of sense, in the organs of respiration and circulation,—in those of mastication and deglutition, of digestion and chylification,—of urine and generation; thus forming five distinct orders of inflammatory diseases, in each of which are included several genera.

In the introductory chapter on inflammation in general, the author judiciously observes, that we must characterize this diseased state, not merely by pain, swelling, heat, redness, and altered function, but especially by the tendency to certain organic changes, both of the fluids and solid texture of the part affected. He briefly but distinctly enumerates in this chapter the causes, varieties, effects, and terminations of inflammation; and not only the general plan of treatment, but many individual remedies, both for the inflamed state and its consequences. In enumerating the causes, (§ 205,) he falls into the error, too common in systematic writers, of under-rating the efficacy of debility as a predisponent cause of inflammation, and representing the inflammatory state as *most easily produced* at that period of life, and in that habit of body, in which there is the greatest bodily vigour, and in which, therefore, *when produced, its symptoms are most striking;* a statement which, we apprehend, is nearly the reverse of the truth.

* "— in the internal parts are found various appearances, which indicate inflammation of the cavity of the fauces, the trachea, the bronchi, the lungs, the stomach, the intestinal tube, or other viscera of the abdomen."

Under the general denomination of Consequences, or, as he terms it, "Exitus Inflammationis," he introduces three long sections, on hydrops, infarctus and obstructio, and scirrhus and cancer; subjects afterwards more fully discussed. We think he is right in taking this method of connecting the general doctrine of inflammation with the most formidable organic diseases, immediately dependent on increased exhalation or perverted nutrition; but his more specific statements on this important part of pathology are by no means precise or satisfactory. He does not state, as we think he might safely have done, that, in order that inflammation may excite any extent of dropsy, it must, in almost every case, be combined either with organic disease impeding the venous circulation, or with obstruction to some of the natural excretions, (generally by the skin or kidneys;) nor does he lay any stress on the *tubercular diathesis*, as the main cause of "infarctus et obstructio post inflammationem," at least in young persons. His enumeration of the causes disposing to such an effect from inflammation, (§ 244,) is a good example of the indiscriminate and careless manner in which alleged causes of disease, internal and external, general and particular, important and trifling, certain and doubtful, are huddled together by many such writers. He says:

"Alimenta ac potulenta peccantia, ingesta acria irritantia, potus coffeæ fortioris, et valde dilutus sed nimia quantitate sumptus, emetica, purgantia, astringentia, venena, aër corruptus, humidus, vita sedentaria, animi affectus ingrati, morbi diversi præter inflammationes, febris intermittentes, *scrofulæ*, syphilis, arthritis, infarctus et obstructiones excitant."*

Perhaps the most instructive part of this discussion is the confidence with which he speaks (like many other continental physicians,) of the efficacy of the different mineral waters and baths, in many cases where there are strong grounds for thinking that organic alterations of texture must have commenced.

We observe, with satisfaction, that, in delivering the history and treatment of the more important inflammatory diseases,—those in which art is more effectual, and error or ignorance more dangerous than any others,—Professor Bene hardly deviates from the course which we consider in this country to be sanctioned by reason and experience. If he is less liberal of bloodletting than those of us who are accustomed to practise on strong and healthy subjects in general are, we must remember that, in the natives of the southern parts of Europe, the effects of the loss of blood appear, from other representations, to be generally greater than with us. There is more ground, we think, for regretting that he is not at sufficient

* "Improper food and drink, irritating acrid ingesta, strong coffee, or weak coffee taken in too great quantity, purgatives, astringents, poisons, bad and moist air, a sedentary life, disagreeable passions of the mind, different diseases, besides inflammations, intermittent fevers, *scrofula*, syphilis, arthritis, produce infarctions and obstructions."

pains to point out the circumstances which demand and justify, and those which contraindicate or forbid, the further loss of blood, in the progress of the different inflammatory diseases; and to avail himself in this important enquiry, as he might have done, of the recent improvements in the diagnosis of thoracic diseases. Nor does he make any mention of the important practical observations of Marshall Hall, Armstrong, Travers, and others, on reaction after loss of blood, and the fallacies thence arising; although the frequent statement made of the "character erethisticus" of febrile disease, and the necessity of caution in evacuations when that state of nervous irritation is well marked, shews that the condition described by these authors had not escaped the author's attention.

Another important objection to the chapters on inflammation of the head and chest is, that he ascribes too confidently to inflammation several diseases of those parts, often connected with a degree of inflammatory action in the first instance, we believe, but not uniformly or necessarily dependent on it, even in their origin; and certainly differing from it widely in their progress, and often in their most proper treatment. Thus, he sets down delirium tremens (hardly known to him, however, by observation,) as a variety of Encephalitis, (t. ii. § 4): he considers hydatids in the choroid plexus, and all varieties of organic disease within the cranium, as effects of inflammation, (§ 5;) he gives a similar opinion, not only of tubercles, but of hydatids, osseous, cancerous, and melanotic tumours in the pleura or lungs, (§ 54;) he seems to suppose vomica of the lungs, without previous tubercles, to be much more frequent than it really is; and, perhaps, a more important error is supposing the emphysema of the lungs to be an effect of peripneumony, whereas we believe it to be certain that this lesion results from bronchitis only; and rarely, as we apprehend, from that, unless combined with occasional spasmodic constriction of the bronchi, as in asthma or pertussis.

We must observe, also, that, in treating of carditis, he lays too much stress on pain and irregularity of pulse, as marking the disease; and says nothing of the dull sound on percussion, and the tenderness at the epigastrium, which in many cases are important aids in the diagnosis.* In this and other cases he does not seem duly aware of the difference of result to be expected from inflammation in different textures; nor does he seem to have clearly perceived the manner in which either dilatation or hypertrophy of the heart supervenes on the effects of inflammation, either of the pericardium or inner membrane.

We are not surprised, after the opinions already noticed, to find scirrhous indurations at the pylorus, or in the intestines, treated as effects of gastritis and enteritis, (§ 90 and 97;) but we should

* The sign of the bruit de soufflet, or bellows-sound, is of too recent date to be noticed by our author. We think it, however, proper to enumerate it in this place, as among the important omissions in the list of signs of pericarditis.

hardly have expected to find ulcers of the mucous membrane of the stomach and bowels described as the sequelæ of abscesses, which have burst and discharged their pus: and, although the author seems well aware of the fact that inflammation most generally affects, in the same subject, only one of the membranes of the alimentary canal, yet he does not appear to have clear ideas as to the peculiarity of symptoms by which the inflammation affecting either the one or the other may, in most instances, be distinguished. (See § 95.) Neither does he appear to have reflected on the peculiarity of the rapidly fatal termination in many or most cases of enteritis, where no considerable disorganization is effected, and no organ immediately necessary to life is injured; nor to be aware of the observations of Armstrong, and other British practitioners, on the advantage which may be derived from full doses of opium in the treatment of enteritis.

In describing inflammation of the liver, he seems hardly aware of the great obscurity of the disease in many cases, where the peritoneal coat is unaffected; and takes no notice of its frequent combination with mucous diarrhœa or dysentery, even in this climate, and still more within the tropics.

He attributes probably too many of the organic lesions of the kidneys to inflammation, acute or chronic; but, in stating the consequences to be apprehended from their chronic induration, he again omits to mention the characteristic appearance of the albuminous urine, of low specific gravity.

He describes the puerperal peritonitis and epidemic puerperal fever, and the gradual transition of the one into the other, fully and carefully, and enumerates, we believe, all the remedies that can afford any prospect of relief: but, although admitting the possibility of the extension by contagion, he never alludes to the simple and (as we firmly believe,) frequently successful preventive expedient, of declining the services of any surgeon or midwife recently in attendance on similar cases.

An omission of considerable importance in the enumeration of inflammatory diseases, is that of arteritis and phlebitis, and their consequences: neither do we observe any mention of those very peculiar and pathologically important internal inflammations, which so often succeed amputation of limbs in which extensive suppuration had been going on.

It is worth while to quote our author's general idea as to the proximate cause of inflammation, which, however vague and unsatisfactory, has the merit of keeping in view the whole of the principal phenomena, and may be preferred to some of the speculations in this country on the nature of the altered action of vessels in inflamed parts, on the simple principle, that "it is better to stand still than go wrong."

"Cum summâ probabilitate pronunciamus, causam proximam inflammationis esse vitam partis unius morbose exaltatam, productam per stimulum morbosum, quem autocratia naturæ removere nititur, qua provocatur propensio peculiaris in metamorphoses dynamico-organicas."* (§ 206.)

Dr. Bene's third class consists of the Exanthematous Diseases, or "Efflorescentiæ Cutaneæ;" and these he arranges almost exactly after the manner, and using the phraseology, of Willan and Bateman, whose works he seems to have carefully studied. His introductory chapter, "De Efflorescentiis in genere," is a favorable specimen of his learning, observation, and judgment; and, by reference to it, we think he might have shortened considerably the details given in many of the succeeding sections. The arrangement being entirely founded on the appearances on the skin, is liable in this, as in all similar works, to the fundamental objection of bringing together diseases widely different in all their most important characters and consequences, (such, e.g., as small-pox and itch;) and, again, widely separating diseases which have, in the most important particulars, strong natural affinities, (e.g. small-pox and measles, erythema and eczema, rupia and impetigo:) but on this point we do not insist at present.

The enumeration of cutaneous diseases begins very properly with erysipelas, and the chapter on that subject is a good one; but the distinction of the genuine erysipelas, (which he regards as one of the febrile exanthemata,) from the pseudo erysipelas, or erythema, (which he considers simply as an inflammation,) is certainly not correctly laid down, when he asserts that the former is rarely excited by a cause acting directly on the skin, (§ 160;) that the latter goes on much more frequently to suppuration and gangrene, (§ 163;) and that the disease excited by punctures in dissection is of the latter kind only, or, as he calls it, Erythema anatomicum, (§ 174.) He says nothing, under either head, of the practice of punctures or incisions prior to suppuration; and does not seem aware, either here or in the discussion on inflammatory diseases, of the important observations of Duncan on diffuse inflammation of the cellular membrane, (always analogous to, but often unconnected with, erysipelas,) nor of Abercrombie and others on inflammations of the diffuse or erysipelatous character attacking internal membranous parts.

The chapters on scarlatina and measles are full and satisfactory, comprising an account of the various forms of these diseases, and judiciously varied practical directions. We are surprised, however, to see no specific mention of wine, as the most important of all remedies in the typhoid form of these diseases. (§ 193, 207.) The account of morbid appearances is altogether omitted in the description of measles, and in that of scarlatina is given in vague terms,

* "With the utmost probability, we pronounce the proximate cause of inflammation to be the life of one part morbidly exalted, in consequence of morbid stimulus, which the autocrateia of nature strives to remove, by which is stirred up a peculiar tendency to dynamico-organic changes."

(§ 189,) without any special reference to the symptoms, or the duration of the different cases in which the different lesions described have been found. We are persuaded that some of the appearances described, particularly the "serum in cavis corporis, jam purum, jam liquido puriformi aut sanguine mixtum,"* have belonged, not strictly to the scarlatina, but to inflammatory affections immediately succeeding it, but often excited by a different cause. This we believe also to be the true pathology of the dropsy succeeding scarlatina, which our author regards as a part of the disease itself, and the very frequent connexion of which with organic lesion of the kidneys he does not seem to have observed.

The chapter on small-pox is elaborate and judicious: he seems to us, however, unnecessarily timid as to the use of opium at the period of maturation; and he does not appear to have observed the combination with bronchitis, and congestion, if not inflammation, in the lungs, which is so common and dangerous with us. He makes a resolute stand, in this and other places, against the untenable doctrine of Broussais, that the debility of typhoid cases is only apparent or sympathetic, and dependent on some local inflammation.

"Verum quidem est," says he, "quod per inflammationem topicam partis imprimis sensibilioris sub decursu variolarum producantur facile turbæ variæ quæ febrim apparenter nervosam referant; utinam omnes tales essent! sed ad lectos ægrorum," (he adds, with perfect truth,) "tristes experiri debemus, dari et febres nervosas genuinas *cum debilitate verâ incidentes;* tales sub exordio non raro mitiorem characterem inflammatorium manifestant, sed ob influxum potentiarum nocentium, ob constitutionem individualem ægri debilem, ob genium epidemiæ regnantis, character nervosus vel nervoso-septicus evolvitur."† (§ 243.)

The accuracy and intelligence of this chapter are indeed such that we cannot fail to be surprised at finding, after a full account of the cow-pox, no further mention of the modified small-pox than the following short and unsatisfactory, if not erroneous, statement:

"Per contagium variolæ genuinæ, et in iis qui variolas genuinas habuerunt, et in iis qui vaccina genuina instructi fuerint, *varicellas generari* observatio repetita docuit." (§ 268.)‡

The account of the other exanthemata, and of the chronic cuta-

* "Serum in the cavities of the body, sometimes pure, sometimes mixed with puriform-fluid or blood."

† "It is true, indeed, that, by the topical inflammation of a very sensible part in the course of variola, various disturbances are readily produced, which apparently belong to nervous fever; and I wish all were such! but we must regret to find at the bed-side genuine nervous fevers presenting themselves with true debility; such, in the commencement, not unfrequently manifest a mild inflammatory character; but, from the influx of hurtful agents, the feeble constitution of the patient, and the nature of the reigning epidemic, put on a nervous or nervoso-septic character."

‡ "Repeated observation has taught that varicella is generated by the contagion of variola, both in those who have had genuine small-pox, and in those who have had genuine vaccinia."

neous diseases, seems to be so much taken from the writings of Willan, Bateman, and Biett, as to call for no further observations.

The fourth class of diseases, occupying the whole third volume, is entitled "Excretiones Morbosæ," and consists of two orders, the Profluvia and Retentiones. This arrangement we think a good one; but his first division of each order consists of Profluvia cruenta, and Retentiones cruentæ; under the first of which heads we have the different hemorrhages, including Menorrhagia, (here termed Metrorrhagia;) and under the last we have not only the Retentio catamenialis and R. lochiorum, but also Retentio epistaxis and R. fluxus hæmorrhoidalis: the two last of which, although properly marked as causes, or as sudden changes of disease, are not correctly entitled diseases; and, although we may admit that hemorrhages, when independent of organic disease, may be ranked along with Profluvia more properly than in any other class, yet those most frequent cases of hemorrhage, which are merely symptomatic of other diseases, ought certainly to be carefully distinguished and separated from the others; which is not done in the work before us. For example, in the account of Hæmatemesis, (§ 39, t. iii.) we are informed that it may result from injury, from poison, or ulceration; that it may be excited without breach of the mucous surface, "per saburram, biliosam, vel verminosam," (which we think doubtful;) by violent exertion, by anger, by suppression of the catamenia or of hæmorrhoids; by tumours of the liver, spleen, or uterus; by sedentary life, by long-continued grief, and by intermittent fever; without any such precise directions as may enable us to discriminate the different cases, which, in their whole pathology, in the prognosis, and practice applicable to them, are so widely different. Thus, also, we have a laboured and indiscriminate enumeration (t. iii. § 29,) of all manner of predisponent and exciting causes, both for the "pneumonorrhagia activa, sthenica," and the "pneumonorrhagia passiva, asthenica;" from which it would be very difficult to extract what is the simple and precise statement of fact, that hæmoptysis is a common effect of excitement of the circulation, in those who have either tubercles or other tumours in the lungs, deranging the circulation there, or ulcers there, or organic disease at the heart; that it is rare when no such organic lesion exists, but sometimes occurs, in young persons, from mere plethora, sometimes as a part of the bloody effusions in purpura or analogous diseases, and also as a vicarious hemorrhage in amenorrhœa.

The true Profluvia are ably and judiciously treated; the chapters on diarrhœa, dysentery, and cholera (both the common and the epidemic species), shew the author to be a careful and candid practitioner; open to conviction on all points, but laudably distrustful of extreme opinions, or exclusive practice of all kinds. In this, as in many other parts of his work, however, we regret that, amid the variety of remedies which he mentions, he does not express himself more decidedly as to those (few, we take for granted, they must be,) on which he really relies in practice.

In regard to the epidemic cholera, (which was very extensively fatal in Hungary, and the rapid extension of which could not, as he thinks, be in any degree explained by contagion,) there are three statements here made, which strike us as important, because they appear to be founded on careful observation: first, that the fumigation by chlorine was used to such an extent, that the manufacturers could hardly supply the demand, until experience shewed that it was really ineffectual, and that "non pauci vapore chlorii continuo circumdati in morbum inciderunt et occubuerunt,"* (§ 145;) secondly, that cold drinks during the disease were peremptorily prohibited by the medical men, until "observatio docuit, quod ægri non pauci, pharmacis omnibus abjectis aquam frigidam hauserint, et feliciter convaluerint;"† after which, cold drinks, and even ice, were freely given, and that under their use reaction frequently took place; a fact, the knowledge of which,‡ in the event of a return of the disease, if unavailing for the cure, may at least be of use in sparing the patients much unnecessary suffering;—thirdly, that the remedy which appeared most effectual, under the author's own observation, in rousing from the state of collapse, was the cold affusion, or even application of ice, to the head and upper parts of the body; while warmth, in one form or another, was assiduously applied to the feet and legs, or even as high as the middle. (§ 155-157.)

Every one who has attended carefully to the subject must, we think, be gratified with the simplicity and good sense of our author's observations on the alleged efficacy of particular remedies: when alluding particularly to the Indian practice of bleeding, calomel, and opium, in cholera, he observes:

> "Summa cum probabilitate choleræ illæ fuerunt tantum mitiores, quales etiam apud nos sanantur; sed in morbo intensiore therapia difficilior est, et pro singulo casu individuali debet therapia rationalis per ingenium medici determinari."|| (§ 149.)

The order of Retentiones demands few observations. Under the head of jaundice, less pains are taken than we should have thought desirable to discriminate the different varieties, and connect their diagnostic symptoms with the appearances on dissection; and hardly any mention is made of the rapidly fatal termination by coma, seen in a few cases of jaundice; especially, we believe, in

* "— not a few persons, continually surrounded by the vapour of chlorine, were attacked by the disease, and sunk under it."

† "Until observation shewed that not a few patients, all medicines being abandoned, drank cold water, and happily recovered.

‡ An observation corroborated by the experience of Dr. Shute, of Gloucester, and many other British practitioners.—Ed.

|| "In all probability, those cases of cholera were only of the milder kind which admit of cure among us; but, in the intenser forms, the treatment is more difficult, and a rational plan must be determined on for each case by the judgment of the physician."

those which depend on absolute suppression, or what our author calls "a paralytic state" of the secretion of bile.

The two last genera in this order are Urolithiasis and Arthritis; and, although the account given of both is able and satisfactory, yet there can be no hesitation, we think, about pronouncing both to be misplaced. Gout should certainly be treated among the inflammations, although in a section appropriated to those which have a specific character.* There is a certain degree of advantage, no doubt, in treating of gout and gravel in connexion with each other; but we can hardly recommend that the latter disease should be placed there likewise: it must, at all events, however, be considered as depending on *alteration,* not on *retention* of the excretion. And that there is a notable deficiency in Dr. Bene's work, in his having no order of diseases dependent on depraved secretions, we think obvious, not only from this erroneous position of the Lithiasis, but likewise from his having actually no place for dyspepsia; certain of the symptoms of which only, e. g. gastrodynia and vomitus, are treated separately, and in widely different parts of his system.

The fifth class of diseases, occupying the whole of the fourth volume, is called the Cachexiæ; but he uses this term in a much wider sense than Cullen did, applying it to all chronic diseases attended with paleness of the countenance, flaccidity of the general habit, weakness, and emaciation; and divides the class, after Raimann, into three orders. The first consists of diseases in which, as he expresses it, the organs of reproduction (or rather of nutrition,) in some one texture of the body are in fault; and here he includes syphilis, as appertaining chiefly to the skin; scrofula and struma (i. e. bronchocele,) as appertaining to the lymphatic system; and rickets, to the bones. The second order consists, as he says, of diseases in which either the blood or secreted fluids are chiefly concerned, and here he treats of scorbutus, of chlorosis, of cyanosis, of what he calls Cacochymia mucosa, (which we should have thought sufficiently discussed under the Profluvia,) of worms, of dropsies, and of Pneumatosis; including under this last term cases of general (not pulmonary) emphysema, of tympanitis, globus and flatulent colic, &c.:—and the third order, "Cachexiæ cum Emaciatione," includes tabes and atrophia infantum, phthisis pulmonalis, laryngea, and what he calls Marasmus senilis.

It is obvious that there is, at least in the first two of these orders, as little attempt at a natural or pathological arrangement of the diseases as in any of the other classes; but several of the subjects are treated with much learning and judgment. The article Syphilis

* It is worth notice, that the author does not seem aware of the much greater influence of certain liquors than of others,—particularly of fermented than distilled liquors,—in producing gout, nor of the degree of efficacy ascribed by many to antacids, (in combination with the bitters which he recommends,) in arresting it. Neither does he seem to have had much experience of the Vinum Colchici; his dose of which, during the paroxysm, is only gtt. xv. twice or thrice a day.

is elaborate and accurate; but we believe few British practitioners will think that he has satisfactorily disposed of the question of the non-mercurial treatment of the disease, such as we now see it in Britain, by the following remark:

"Adferuntur quidem observationes multæ medicorum Anglorum, Gallorum, et Germanorum, quibus probare nituntur, contra ulcera syphilitica hydrargyrum non esse necessarium. Observationes virorum eximiorum in dubium haud vocamus, sed illud nobis maxime probabile est, ulcera quæ consectariis nullis subsecutis sine hydrargyro sanata sunt, non fuisse vera syphilitica."* (T. iv. § 31.)

The answer to this we apprehend must be, that, if so, the ulcera "vera syphilitica" must be so rare in Britain at present as to demand very little attention, in comparison with other complaints springing from the same origin, and following the same general course, as to the textures of the body they successively affect.

Dr. Bene is well aware of several of the dangers to be apprehended from mercury in these diseases, and, among others, mentions ulcers in the mouth and fauces, closely resembling the syphilitic, (§ 29;) but he does not seem to regard affections of the bones as in any case or degree resulting from the use of mercury, (§ 36:) he says nothing of syphilitic ulcers, or other affections, being peculiarly modified by the scrofulous habit, and being, in scrofulous cases, injured or renewed by the use of mercury; and, although he says he would not recommend the "cura magna" by profuse salivation to persons "dispositione nervosa vel phthisica præditis,"† yet he is so far from regarding scrofulous disease as a strong or general contraindication to the use of mercury, that, while he gives a proper caution against proceeding immediately to the mercurial treatment, when the syphilis is complicated with febrile or inflammatory disease, with scurvy, or with any important nervous disorder, he goes on to say, "Subinde complicatur syphilis cum aliis morbis, ut *scrofulis,* rhachitide, infarctibus abdominalibus, qui, licet diversa symptomata, causas et exitus habeant, tamen *therapia eadem,* quæ contra syphilidem indicata est, removeri possunt."‡ (§ 38.) He appears to have seen trials, both of the non-mercurial English treatment, and also of Hahnemann's infinitely small doses, (which is, at least, as fair a trial of the *vis naturæ medicatrix* in these complaints,) but asserts positively that, in Hungary, these methods have failed: and, so strongly impressed is he with the idea of mercury acting strictly on the footing of an antidote to the syphilitic

* "Many cases are adduced by the English, French, and German physicians, by which they have endeavoured to prove mercury to be unnecessary in the treatment of syphilitic ulcers. We do not doubt the observations of celebrated men; but to us it appears in the highest degree probable, that the ulcers which have been cured without mercury, without any after-consequences, were not syphilitic."

† "Of a nervous or phthisical constitution."

‡ "Syphilis is occasionally complicated with other diseases, as scrofula, rickets, abdominal infarctions; which, although their symptoms, causes, and terminations are different, may be removed by the treatment indicated in syphilis."

poison, that, in all cases where the system is distinctly infected, and the disease appears inveterate, he strongly warns his readers against stopping or diminishing the remedy when salivation is commencing, and again resuming it, so as never to affect the mouth strongly. By these means, he says, the disease is ineffectually opposed, while the noxious effects of mercury are allowed to take place, and the case is rendered more complex. In all such cases, therefore, he recommends a "salivatio justa quantitate," which he seems, in general, to keep up for several weeks. (§ 42.) In all these respects, his inferences from experience seem to us materially different from those now generally drawn in this country; but whether this difference can be ascribed to inaccurate observation, or whether the poison of syphilis is really somewhat different, or the complication of it with scrofula less formidable in that country than in this, we do not pretend to judge.

The "cura magna" of mercurial inunction, causing ptyalism to the extent of three pints and upwards in the day, and continued at least four weeks, (§ 41,) is evidently his favourite remedy, in all tolerably healthy persons, in whom secondary symptoms have appeared: but he gives a list (§ 46,) of about one hundred vegetables which have been used as substitutes or auxiliaries; among which we must confess are many, (e.g. the Xanthoxylum fraxineum, Ziziphus trinervium, Zigophillum fabago,) wholly unknown to us; and he speaks highly of the decoction of Zittmann, "quo solo et lues syphilitica perfecte evoluta, et neglecta, et hydrargyro minus congrue tractata sæpe felicissime et brevissimo tempore sanatur."* This decoction is prepared after two formulæ, a stronger and a weaker. Of the former, a quart is taken, hot, in the course of the forenoon; and of the latter, an equal quantity, cold, in the afternoon. Sarsaparilla is the main ingredient in both, but in the former there is a quantity of calomel, of cinnabar, (which, as the liquor is strained, we should suppose must be ineffective,) of alum, and senna; the latter contains only some aromatics along with the sarsaparilla. The "cura" in this case lasts eleven days, but these medicines are taken only during eight of them, the first, sixth, and eleventh being occupied by full purging with jalap and calomel. The patient is confined to bed during and for some days after this discipline. The course is sometimes repeated; but "subinde nec repetitum decoctum votis respondet."† We can hardly doubt that these complaints are treated in a safer as well as simpler manner with us; but it would surely be very desirable to have accurate observations, and, if possible, numerical statements, to illustrate both the usual varieties of the disease in Hungary and here, and the comparative effects of this and the British practice.

The chapter on scrofula is short, and, considering that there had

* "By which alone both syphilitic lues, perfectly evolved, and neglected, and improperly treated by mercury, is often most happily cured, and in a very short space of time."

† "Sometimes even the decoction, when repeated, is ineffectual."

been no previous discussion on the scrofulous variety of inflammation, is in several respects unsatisfactory. The usual marks of the scrofulous diathesis are enumerated, but chiefly as applicable to the case of children; and the gradual degeneration, which may certainly take place, from the state of perfect health, to that in which scrofulous disease is easily excited, and which, we believe, may be produced at almost any period of life, by the long-continued action of debilitating causes, is not stated nor illustrated. The influence of hereditary tendency, of improper diet, of impure air, of sedentary habits, and of previous disease, in thus modifying the human constitution, are briefly stated, (§ 50); and these are really, we believe, the most powerful causes; but something should also have been said of climates and seasons; something of mental depression or anxiety, and more of the use of strong liquors, than is implied in the mere enumeration of distilled spirits, along with potatoes, among the articles of diet which may favour the scrofulous tendency.

The disease is regarded as chiefly seated in the lymphatic vessels, and accurately described as it appears in the external lymphatic glands: but there is an enumeration of the diseases depending on scrofulous internal affections, in all the cavities of the body, (§ 49,) which opens a wide field for pathological discussions, and practical admonitions, subsequently almost entirely neglected. The frequent occurrence of chronic inflammations, in connexion with distinctly scrofulous disease, is merely stated, (§ 51;) but there is no minute description of scrofulous tubercles, nor enumeration of the textures in which they form, nor statement of the other morbid appearances usually attending them, nor discussion of the question, how far the inflammatory appearances, so frequently combined with them, can be regarded as indicating their exciting cause, or in what circumstances these are to be held to be merely their effects.

The importance of a tonic regimen in correcting the scrofulous diathesis, and, on the other hand, the frequency of inflammatory symptoms, demanding a somewhat antiphlogistic treatment, are fairly stated, (§ 52 and 53;) but we think our knowledge of the morbid changes usually found in scrofulous cases would have warranted some more specific statements as to the use of these last remedies than the following:

"Per observationes circa ægros convincimur, in scrofulosis adesse diathesim phlogisticam peculiarem, et non raro in membrana mucosa ventriculi et tubi intestinalis evolvitur inflammatio; dum eam observamus, exhibemus initio emollientia, externe quoque applicamus cataplasmata emollientia, quibus haud sufficientibus, statu phlogistico intensiori reddito ad hirudines recurrimus, caute tamen, bene gnari, quod per hæmorrhagiam ultra justos limites continuatam morbus scrofulosus universalis incrementa nova capiat."* (§ 53.)

* "We are convinced, by observations made on the sick, that there is a peculiar phlogistic diathesis in scrofulous subjects, and that inflammation is not unfrequently

In regard to the use and degree of efficacy of the tonic regimen, of baths, of alkalies, acids, various alteratives, and iodine, in correcting or opposing the scrofulous tendency, we find some sensible and not exaggerated observations, but no novelty.

The chapters on bronchocele and rickets are more satisfactory, we think, than that on scrofula; but, passing over them, and also several of the cachexiæ thought to depend on a morbid state of the blood, or secreted fluids, we go on to notice the chapter on dropsies; and shall only remark on this part of the arrangement, that, if it be, as we certainly think it, improper to rank dropsies in the same order with scurvy and with cyanosis, it seems equally inconsistent with good arrangement to rank acute hydrocephalus as the first of the dropsies; more particularly as serous effusion, directly consequent on inflammation, had been fully discussed formerly, and as the only practice recommended for the hydrocephalus is the vigorous use of antiphlogistic remedies, and especially the "sanguinis evacuatio nunquam negligenda." Two important objections occur to us to the treatment here enjoined for acute hydrocephalus, (§ 100.) 1. The bloodletting is advised to be from the arm only, "in ægro adultiori habitu plethorico prædito." Now we have seen so many examples of the decided efficacy of general bloodletting, and of the comparative inefficacy of leeching, in children even of feeble habit, that we are confident in recommending the former, in all well-marked cases beyond the age of four or five; and regard it as contra-indicated, or likely to fail, in this as in many other cases, not where the habit of body is feeble, but only when the slow, gradual, and insidious progress of the disease indicates that the action on which it depends is materially different from active inflammation. 2. He seems to use calomel chiefly with a view to its constitutional effects, and does not seem to be aware of the decided advantage, seen in many cases of at least threatening hydrocephalus, even at a later period than that to which bloodletting is adapted, from full and repeated purging.

The section on hydrothorax we do not think satisfactory. It is not stated with sufficient distinctness, that the serous effusion is in by far the greater number of cases only an effect, (often one of the last effects,) of disease, either of the heart or lungs, of such extent as to be highly dangerous, even if this particular result should not follow. Hardly any of the symptoms enumerated (§ 109) are characteristic, and, although he assigns the proper cause for the starting from sleep, he is certainly incorrect in saying, that the fluctuation of the fluid is ever audible in a case of simple hydrothorax: he is not sufficiently precise in his statement as to the dull evolved in the mucous membrane of the stomach and intestinal tube: observing that, we exhibit in the beginning emollients, and apply emollient cataplasms externally; if these do not suffice, the phlogistic state becoming more intense, we have recourse to leeches, cautiously however, well knowing that, by bleeding carried beyond its just limits, scrofula is aggravated."

sound on percussion, and the absence of the respiratory murmur, being in some cases of hydrothorax (we do not say in all,) distinctly observed to change their seat, on changing the position of the body; nor does he explain, as clearly as he might have done, the means by which we are to judge whether the serous effusion is complicated chiefly with disease of the heart, with bronchitis, œdema or emphysema of the lungs, or with the effects of previous inflammation in the lungs themselves; nor is there any minute specification of the different organic lesions of the heart and great vessels which may obstruct the flowing blood, nor of the means of distinguishing them. On all these subjects we are willing to admit, that many recent works exhibit a minuteness of diagnosis not easily attainable, nor easily applied to any useful purpose, if attained; but it is in vain to deny, that knowledge of several of these points, where it can be procured, is occasionally very important in practice. We willingly admit, however, that our author shews himself to be well acquainted with all the useful remedies, and with most of the principles which assist us in their selection, (§ 112.)

The sections on anasarca and on ascites we think less open to objection than that on hydrothorax; and in particular the occasional effect of inflammatory action in producing these effusions is fairly admitted, without being too exclusively dwelt on; but here, as elsewhere, we must complain of the indiscriminate enumeration of causes, predisponent and exciting, internal and external (§ 95 and 116.)

The Cachexiæ cum emaciatione are, almost exclusively, a supplement to the chapter on scrofula. Under the head of Tabes there is a description of gradually increasing emaciation and debility, passing on to hectic fever, and then an enumeration of the various modes in which it may be conceived that the natural process of nutrition of the body may be so impaired, and that these consequences may follow, (§ 128;) great part of which seems to us to have no foundation in practical observation, but in which organic diseases of the different abdominal viscera are properly included. This chapter we regard as an illustration of the difficulty of incorporating the older distinctions of diseases, founded on mere observation of their symptoms, with the more accurate knowledge of the organic lesions attending or producing them, which modern pathology has obtained. We would further observe, that, in speaking of ulceration of the bowels as one of the causes of hectic fever, and in his distinction of the bilious and mucous hectic from the nervous hectic, he does not seem aware of the frequent occurrence of congestion of blood, even of inflammation, and ulceration in mucous membranes, as a consequence, rather than a cause, of extreme emaciation and debility, and of the illustration of this principle from what is seen in fasting animals.

The chapter on Atrophia infantum is an account of the Tabes mesenterica: our only objections to which are, that it describes the disease as following a more uniform course than it generally does; and that it contains no minute description of the changes that take place in the mesenteric glands during the disease, nor of the lesions of other parts which so often accompany it.

The chapter on Phthisis affords ample evidence that, although the author would recognize the disease perfectly, and treat it judiciously, in a *great majority of cases,* yet he is not intimately acquainted with the changes which the structure of the lungs undergoes, nor with the surest indications of those in the living body; and in *a few cases* this want of information must be injurious. He describes four species of the disease; in the first two of which, (P. nervosa and P. catarrhosa,) the substance of the lungs is unaffected, which therefore are modifications of bronchitis, the first without, the last with, copious expectoration. His third species is the Phthisis apostematica, which he seems to regard as the only one really consequent on inflammation of the lungs, and in which he supposes one or more vomicæ to form in the lungs, not preceded by tubercular deposits. Lastly, his Phthisis tuberculosa includes all the cases generally regarded as belonging to the true phthisis. This he seems to regard as occurring only in those who have the usual marks of the phthisical tendency, or tubercular diathesis, and he describes the symptoms in them as constituting four stages: the Stadium incubationis, S. inflammatorium, S. suppurationis, and S. colliquationis. There is a supplementary chapter on Phthisis laryngea and trachealis, which does not demand any special comments.

He makes no mention, in treating of the Phthisis catarrhalis, of the emphysematous state of the lungs, which is that complication, according to our experience, which most frequently makes the bronchitis of young persons take the form of phthisis: he does not seem aware of the extreme rarity, according to all recent experience, of the P. apostematica, such as he describes it: he says nothing of the case, by no means uncommon, when the P. tuberculosa is so general over the lungs as to be fatal in the first stage, almost before hectic has formed, certainly before there is suppuration or ulceration, or when these are of trifling amount, and where the symptoms are those of somewhat chronic pneumonia rather than of phthisis, and would seem to come under his head of P. apostematica rather than tuberculosa. He hardly states the question of the origin of tubercles, or rather of the possibility of their originating in different ways, and of one mode of their deposition being counteracted by antiphlogistic treatment: he does not notice the remarkable variety of the symptoms of the disease as occurring in old or young persons; nor does he illustrate the incessant operation of the main external causes of the disease by any statistical statements.

Again, his account of the symptoms yielded by auscultation in phthisical cases (§ 141,) seems to us not only meager but incorrect. He says that in the crude state of tubercles, in the part of the chest where they lie, "nullus percipitur strepitus respirationis,"* without remarking, that this is only when a certain number of them have coalesced: he makes no mention of the *gargouillement,* or *râle caverneux,* as going along with resonance of the voice when a cavity has formed; and he seems to think that the metallic tinkling is to be heard whenever a tubercular cavity (not mentioning its size) is only partially emptied; and that the ægophony (vox tremula similis voci caprarum) is characteristic of the state of pneumothorax supervening on phthisis. (See the last sentence of § 141.)

It is right to state, however, that the prophylactic measures which he advises appear quite judicious; that he seems well acquainted with all the remedies by which the disease may be restrained or palliated; and that he puts the question of the possibility of recovery on its true footing, by stating that there is nothing essentially malignant, or incapable of favorable termination in the existence, or even in the suppuration and ulceration of tubercles; but that their fatality depends simply on the extent and frequency of the successive deposits, and on the extreme difficulty of correcting the tendency to them, after they have once commenced. (§ 143 to 147.)

The sixth class of diseases, occupying the whole fifth volume, consists of the Neuroses; and we think it obvious, that the diseases he has referred to this class form a much better marked natural family than those described by Cullen under the same title. They are divided into Dolores, Spasmi, Debilitates, and Vesaniæ; and these orders are also, we think, correctly marked out, although the term debilitates, applied to apoplexy, palsy, syncope, or animi deliquia, and asphyxia, is objectionable, and some of the genera in this and other orders seem to us to be misplaced.

The general and obvious distinction of the functional and organic diseases of the nervous system is clearly pointed out in the preliminary chapter, under the quaint expression Neurosis sine materia and Neurosis cum materia; there is likewise a fair statement of the importance, and at the same time of the difficulty, of the distinction of idiopathic from symptomatic affections of the nervous system; and we must admit that, however desirable it may seem, it is hardly possible, in the present state of our knowledge, to assume either of these distinctions as the groundwork of a nosological arrangement. There is likewise, in this preliminary chapter, a judicious and useful summary of the various means of treatment which may be employed in nervous diseases; and the general indications for them are drawn, not so much from the nervous affections themselves, as from the state of the circulation, digestion, and general health and strength, with which they may be combined. (§ 6-10, t. v.)

* "No respiratory murmur is heard."

But, in the discussion of nervous affections in detail, we meet with various errors and deficiencies; some of which are perhaps inseparable from the formal mode of distinguishing and treating them which is here adopted. Admitting that it is extremely difficult to form genera of nervous diseases, at once grounded on true pathology and available in practice, we must still observe, that, when single symptoms, common to so many diseases, as cephalalgia and rachialgia, are formally treated as separate diseases, and all their causes enumerated, not only a great deal of repetition is inevitable, but due weight cannot be given to other and often more diagnostic symptoms, nor to distinctions founded on the nature and effects of diseased actions.

We shall notice some objections that occurred to us in looking over these chapters.

Under the head of Cephalalgia, it is stated that headachs, connected with intermittent fever, may be very intense, but intermitting, and may be cured by bark; and, again, that headach may proceed from syphilis, and be cured by mercury; but it is not stated that intermitting headachs, although totally unconnected with ague, may be successfully treated nearly in the same way; and that headachs, not strictly venereal, if connected with disease of the bones and membranes, may often be relieved by mercury or analogous remedies. Under the head of Rachialgia he describes, although very imperfectly, the morbus dorsalis, as if it were a consequence of affection of the spinal chord, "in morbo diutius durante ob processum dynamicum abnormem exoriuntur inflammationes lentæ in vertebris,"* &c. (§ 22;) which, we apprehend, is hardly ever the real cause of the changes.

Under the head of Neuralgia, we observe no mention of those stitches in the sides, or in various parts of the integuments of the trunk of the body, which often deserve the name of neuralgia rather than any other; and neither here nor under the Rachialgia is mention made of the tenderness over the vertebræ next the origin of the spinal nerves which may be affected, as a mark certainly sometimes aiding the diagnosis of such nervous pains. The term neuralgia is not applied here to any pains of internal parts; nor is it stated, as distinctly as we think it might have been, in the chapters on angina pectoris, on gastralgia, and enteralgia, that some of the severest pains ever felt in internal parts seem to be strictly of the same character.

That, under the title of Gastralgia and Enteralgia, our author puts together cases which have no pathological connexion, and which it is truly important to distinguish in practice, appears distinctly from the following enumeration of the morbid appearances

* "When the disease is of longer continuance, from the irregular dynamic process, slow inflammation takes place in the vertebræ," &c.

found in the latter case: "Reperiuntur non raro indurationes in una vel pluribus partibus tubi intestinalis, subinde emollitiones morbosæ, in uno loco coarctationes, in alia dilatationes, exulcerationes jam superficiales, jam profundiores, subinde perforationes tubi intestinalis,"* (§ 58:) but, while this statement is made, there is no specification of the means of distinguishing these different cases from one another, or from the case where "dolores intensissimi excruciant ægrum tantum ob sensibilitatem morbose exaltatam."†

We doubt the propriety of placing Odontalgia among the neuroses; and still more the propriety of treating of the various complaints connected with the teething of children under the same head as the toothach of adults, as is done here. Indeed, several of these complaints are so widely different from each other, that it seems obvious that the cutting of the teeth should be considered as their exciting cause merely, not as their nosological character.

The Spasmi form a natural family of diseases, so easily distinguished from all others that there can be no difficulty as to their being treated together; and our author has certainly done right in excluding some of the genera placed here by Cullen, particularly the diarrhœa and diabetes; but we think it clear that the hypochondriasis, notwithstanding what is so often said of its alliance with hysteria, should be ranked with the Vesaniæ, not with the Spasmi: and equally clear that both incubus (nightmare) and angina pectoris belong to the Dolores, rather than the Spasmi. If any affection of the heart belong to this class, it is clearly as Cullen taught, the palpitation, not the pain, which in many cases is more correctly described by the term Syncope anginosa.

The true spasmodic diseases, in which the muscles of voluntary motion and of respiration are concerned, are fully and accurately described; and the practice best recommended by experience for their cure or relief is, we believe, fairly and judiciously stated. The hydrophobia seems a favourite subject: no reliance is placed on any means of prevention or cure, except excision or cauterizing; but it is stated that the best preventive recommended is the powder of belladonna, given twice a day, in small doses, gradually increased till the head is affected, and the affection maintained for some days; and that the remedy, after bleeding and mercury, which has seemed to have most effect, is the powder of cantharides, in gradually increasing doses given every two hours, till some of its irritant effects are observed.

The author does not seem aware of the degree of advantage which many practitioners in this country have thought they have

* "Indurations are not unfrequently found in one or more parts of the intestinal canal; occasionally morbid softening, contractions in one part, dilatations in another, ulcerations deep or superficial, sometimes perforations."

† "The most excruciating pains affect the patient solely from morbidly exalted sensibility."

4v18

obtained from the continued use of purgatives, in tetanus, hysteria, and chorea; and, in the last case in particular, he seems to think that the advantage from purging is confined to the cases where morbid matters seem to have accumulated in the intestines, and are evacuated under their use, (see § 126;) which, we are satisfied from observation, is by no means the case. We apprehend that great part of the beneficial effect of continued purging in the chronic nervous affections is to be ascribed to their acting as *derivants* or counter-irritants on a large scale.

Under the head of Asthma and Pertussis there is a serious omission, in no mention being made of the emphysematous state of the lungs, naturally consequent on both these diseases, and often acting as the chief cause of other more dangerous sequelæ.

The next order, the Debilitates, is of much more questionable propriety. It includes all the varieties of apoplexy and palsy, syncope (or Animi deliquia), and asphyxia, including the A. "ab aëre mephitico," which is properly a case of poisoning, that "per congelationem," and that "a fulmine." Several of these affections are, in point of pathology, as widely different as possible, and several of them being the immediate effects of injury or violence, are, we think, more properly treated separately, and premised to any account of diseases, than ranked as varieties of disease.

Although apoplexy is well described, most of its external causes and varieties enumerated, and the premonitory symptoms and preventive regimen, as well as the treatment, accurately and carefully stated, no mention is made of the well-marked variety, accurately described by Dr. Abercrombie, in which the first attack resembles syncope, and is followed by a short recovery of sense and motion, and then by gradual, profound, and fatal coma; the course of the symptoms accurately resembling those of concussion of the brain attended with extravasation, and the appearances on dissection being uniformly extensive effusion of blood. We must observe, also, that the account of the morbid appearances after apoplexy (§ 174,) is vague and imperfect: there is no attempt to connect the very various appearances with variety in the symptoms or progress of the disease; there is no description of the changes which follow the effusion of blood, in cases not quickly fatal; nor any mention of the diseased state of the vessels within the cranium, so often seen to accompany, and in part to explain, the effusion of blood: nor are we satisfied with the specific character assigned to the "Apoplexia asthenica," demanding, as the author thinks, the stimulating treatment, which character, it is said, the disease "habet ab exordio in ægris exhaustis, cachecticis, per evacuationes nimias in hunc morbum conjectis."* For it is certain that the true sanguine apoplexy, admitting of relief from evacuations, may

* "—has from the beginning in exhausted and cachectic patients, in whom the disease is brought on by excessive evacuations."

occur in the most weakly and exhausted subjects; and we doubt whether truly apoplectic symptoms are ever the effect of excessive evacuations, excepting in the case, accurately described by Abercrombie, by Gooch, and Marshall Hall, (none of whom are quoted on this point,) of young children falling into a state of stupor after severe diarrhœa; in which case, the coldness of the surface, feebleness of pulse, and absence of palsy, are important diagnostics, not here specified.

In regard to the treatment of palsy, we are not surprised at the statement, "methodus antiphlogistica in casibus tantum paucioribus proficua est;"* but we should hardly have expected an experienced practitioner to add, (without any distinction of the cerebral from the partial or merely muscular palsy,) "in longe pluribus, ob characterem totius morbi asthenicum, remedia stimulantia jam mitiora jam fortiora *necessaria* sunt,"† (§ 184;) because, without absolutely denying the specific agency of certain stimulants in palsied nerves or muscles, we cannot but regard their use as often hazardous, and their efficacy as generally slight, and look on the recovery from the usual form of palsy as chiefly the work of nature, and one to which she is perfectly competent, in cases where the general health has been previously good, and where the lesion of the nervous system has not been originally great, and has been prevented, by the proper remedies, from extending itself.

Having already protested against the various cases of syncope and asphyxia being classed along with apoplexy and palsy, we shall only further observe on these affections, (the nature of the danger in which seems to be, in general, correctly apprehended by our author, and the treatment of which is, we think, very judiciously laid down,) that, if the effect of extreme cold, and of lightning, are properly treated under the head of asphyxia, so also ought the effect of extreme heat, or *coup de soleil,* which is only mentioned as one of the causes of apoplexy, (§ 173.) But, in fact, the effect of all these is more analogous to *concussion* of the brain than either to apoplexy or suffocation, and they ought to be studied in connexion with the different cases of concussion which are, in the first instance, unattended with lesion of the structure of the brain.

There can be no question as to the propriety of forming a distinct class of the diseases last treated by our author, the Vesaniæ; and the chapter on this subject is an excellent one, indicating both much reading and habitual observation and reflection. The metaphysical statement of the aberration of the mental faculties does not appear to us satisfactory; and we perceive little information to assist us in the decision of those difficult questions, often occurring in courts of law, when the exact line of distinction between insanity and mere irritability of temper, or eccentricity of character, is to be

* "The antiphlogistic method is only advantageous in very few cases."

† "In by far the greater number, on account of the asthenic character of the whole disease, milder or stronger stimulant remedies are necessary."

traced; but the history, the chief causes, the varieties, and the proper treatment, physical and moral, of these lamentable diseases, are fully and judiciously stated and illustrated. The general rules laid down as to the construction and economy of lunatic asylums (§ 217-18,) are worthy, we think, of all commendation. We must add, however, that the morbid appearances found after insanity are mentioned (§ 215,) very briefly and somewhat vaguely, and no mention is made of peculiarity of form of the bones of the head, certainly obvious in many such cases. The only objection that struck us to the treatment recommended is, the unnecessary and even dangerous timidity evinced by the author as to the use of opium in delirium tremens, (§ 235;) which we can only explain by supposing him to have had, fortunately for his country, little opportunity of observing that form of insanity.

We have looked over the therapeutical parts of the work with the special view of observing any remedies or modes of practice which are unknown in this country, without further success than finding a number of vegetables named as fit for fulfilling various indications, with which we are not acquainted, (e. g. among diuretics, the Cainca, the Ballota lanata, and Ononis spinosa;) but the enumeration of individual remedies is in all cases so copious, that we often feel desirous of more decided recommendations as to their comparative efficacy, and the proper selection to be made among them.

The great fault of the work, however, which forbids our anticipating for it an enduring or extended reputation, is its not being sufficiently grounded on pathology. The author is not unacquainted with the best modern authors on morbid anatomy, but he has not made the proper use of their labours; and it is important to observe in what manner he has failed to turn them to proper account. He has attempted, as we have already observed, to ingraft the more accurate modern distinctions of morbid appearances on the older distinctions of diseases, drawn from their external symptoms only; and the attempt, instead of simplifying, has only given additional complication, both to the discussion of individual diseases and to the general arrangement of the subject. The proper use to be made of the more accurate morbid anatomy is to correct the histories and remodel the distinctions and arrangements of diseases from their foundation,—to separate those distinctions which are essential and uniform, and founded on the nature of diseased actions, from those which are dependent on adventitious circumstances, and therefore liable to continual variation,—and thus to give simplicity and precision to our views of the distinctions among diseases, and facilitate the application of practical experience.

It is true that there are many diseases, into the nature of which morbid anatomy gives us no insight; and that, with respect to all, the information it gives is available for the explanation of the phenomena only up to a certain point: but it always enables us to judge whether a disease, or the fatal event of a disease, is susceptible of explanation in this way or not. It is always one of the elements by which we are to judge of the nature and true distinctions of diseases; and those cases in which nothing is seen to account for the fatal event, are just as valuable, in this view, as those where it is most satisfactorily explained.

It is to the arrangement of acute diseases that these observations are most applicable; and it is here that the defects of our author's plan are most conspicuous. We have already stated that, by the method which he follows, cases of several of the strictly inflammatory diseases fall to be considered along with the fevers, as in the case of Febris gastrica, Febris catarrhalis, Febris rheumatica. In like manner, it is plain (particularly from § 98, t. i.) that, under the head of "Febris cum charactere nervoso," he includes cases of decided inflammation of the brain; and we have already remarked that, among his Efflorescentiæ cutaneæ, he has diseases in juxta position which are, in all respects except the appearance of the rash on the skin, the most widely dissimilar.

On the other hand, we find descriptions of diseases essentially identical in very different parts of his work: we have typhoid fever, for example, such as it daily presents itself to our observation, fully described, first among the "Febres continuæ cardinales;" we meet with it again, with such slight complications as are of daily occurrence, under several of the "Febres continuæ compositæ," (see particularly § 108, 116, 123, 135;) and then we have another full discussion of the subject, under the title Typhus contagiosus Europæus; and, lest any cases should escape observation under this last head, it is distinctly stated that what is here described may arise from miasma as well as contagion, and that it is very frequently complicated with local inflammatory affections in all the different textures and organs of the body. (§ 159 *et seq.*)*

Again, it is impossible to read the descriptions of the Febris catarrhalis and the Febris gastrica mucosa, in the first volume, (i. e. of those varieties, there described, in which the fever has the inflammatory, not the septic or nervous, character), and again the description of Bronchitis, (§ 30, t. ii.) and of Dysenteria, (§ 118, *et seq.* t. iii.,) without perceiving that the same diseases are there described. Our readers will at once perceive that the arrangement of the chronic diseases is also completely artificial, and in many instances liable to similar objections. Thus, we have scrofula and

* Indeed, the whole long discussion on the Febres continuæ cardinales, considering that the different forms and complications of continued fever are fully described afterwards, is clearly obnoxious to the ridicule thrown by Broussais and his followers on medical "Ontologists." The term is incorrect, and the ridicule inapplicable, in the case of those pathologists who merely maintain that fever is a *general* disease and distinguished from one of *local* origin; but they are strictly applicable to those who, after describing the history and treatment of different forms of febrile disease, tell us that these are, after all, only *abstract* forms, not actually occurring in practice.

rickets in one order of the Cachexiæ; phthisis and tabes mesenterica in another; diarrhœa, of all descriptions, among the Profluvia; and infarctus and obstructio of all sorts among the "Exitus inflammationis."

Now, the laboured distinctions and arrangements of diseases can be of no use, unless the young practitioner is taught to believe that it is of practical importance for him to know to what part of the nosology which he learns the individual cases that occur to him are to be referred; and, if he believes this, what result can follow from his finding the same phenomena described under so widely different heads, and different phenomena under the same? Nothing, we apprehend, but confusion and consequent irresolution.

We do not mean to deny that there are difficulties in the way of any systematic arrangement of diseases, founded on such knowledge of their pathology as we possess; but we are confident that a much nearer approach to such an arrangement than has been here attempted might easily be made, and would greatly simplify the subject, and prevent many repetitions. In fact, Cullen's Nosology, so far as acute diseases are concerned, is a much nearer approach to a natural method of arrangement; and the subsequent investigations in morbid anatomy ought to be employed, by any systematic writer of the present day, to simplify, not to complicate or mystify, that arrangement.

In particular, we may derive from this source a full confirmation of the expediency of maintaining, even more decidedly than Cullen did, the grand and essential distinction between the inflammatory and the strictly febrile diseases, under which last head we should include, not merely fevers, continued and intermittent, but all the contagious exanthemata, and probably what are usually regarded as certain forms of inflammatory diseases. That this must be really the foundation of any natural arrangement of acute diseases, founded on a knowledge of their whole pathology, appears, we think, from attending to the following points, in which these diseases are remarkably contrasted with one another.

1. The inflammatory diseases depend on causes which are constantly in operation, and therefore occur pretty uniformly in every large community, when long periods of time are compared, undergoing pretty regular variations of frequency, according to climate, season, and modes of life; whereas the strictly febrile diseases depend on the agency of specific poisons, which are frequently absent even from large communities, in extensive districts, or for long periods of time; and again, at other times, attack great numbers within narrow limits of space or time, either endemically at certain seasons and in certain localities, or epidemically at irregular periods.

2. The causes exciting the inflammatory diseases are, in general, external to the living system, and their application is necessarily transient; but, from the time of that application, certain of the symptoms usually continue, until the diseases themselves are fairly developed: whereas, those which excite the strictly febrile diseases have always a certain latent period, indicating that they have been taken into the system, and are silently working within it for some time before their effects shew themselves; and, in most of these cases, the continued operation of a poison, which has been introduced into the body, is further shewn by the discharge of effluvia, capable of acting, in their turn, as a cause of the same disease.

3. The whole history, the terminations, and the sequelæ of inflammatory diseases, and the experience of remedies in them, establish these points,—that they depend on diseased *actions*, which have been excited in the system, but which depleting remedies, if early employed, can arrest, and which, if not arrested, naturally lead to certain alterations of structure, permanent, at least for a time, after the actions producing them have ceased; whereas, the strictly febrile diseases appear, when we reflect on the corresponding parts of their history, on the peculiarity of their typhoid symptoms, and on their critical terminations, to depend on the operation of *poisons*, which have been taken into the system, and which our remedies cannot expel. These diseases therefore, when once excited, must, in almost all cases, run their course; and, although they may be *modified*, cannot be *arrested* by depleting remedies,* nor by any thing acting otherwise than as specific antidotes; and, when they abate, however violent they may have been, and however slight the depletion by which they may have been opposed, unless they have been distinctly combined with local affections, not essential to their nature, they leave behind them no marks of alteration of internal structure.

4. When the inflammatory diseases are fatal, they very generally leave behind them tolerably uniform and characteristic indications of injury to the parts affected, such as, according to known laws of the animal economy, afford an adequate explanation of the fatal event: whereas, the morbid appearances left after the strictly febrile diseases are remarkably various, are comparatively slight, sometimes imperceptible,† and very generally inadequate to the explanation,

* The case of continued fever, cut short by bleeding, vomiting, and purging, is hardly an exception to this principle; because it is a case of very rare occurrence, and because, when it does occur, it seems certain that the amount of evacuation, effectual for the purpose, is often trifling, and therefore that it is not on the principle of depletion that these expedients act.

† We very much doubt whether the exudation either of coagulable lymph, or of pus, such as characterize healthy inflammation, in any internal part, is *ever* the consequence of any of these strictly febrile diseases, if carefully distinguished from the inflammations which frequently supervene upon them; and we are certain that any such strictly inflammatory effusion, *in the course* of these diseases, must be extremely rare. The internal inflammations attending them are in many cases obviously accidental combinations, and in all appear to be of peculiarly modified or specific character.

on these principles, of the fatal event; and therefore coincide with the other particulars already noticed in indicating the noxious operation of a poison introduced into the system, and operating there independently of any inflammation which may either coexist with its action, or even be excited by its own influence.

It is because we are confident that this last statement is a correct account of the general result of experience in these diseases, that we have asserted that the modern improvements in morbid anatomy confirm the propriety and importance of maintaining this line of distinction among acute diseases. We are perfectly aware, that cases occur, in which it may be difficult to observe the distinction with certainty. The law of continuity seems to be so generally observed by nature, that we can hardly point out any two natural objects, however strongly contrasted, towards which a series of intermediate links does not exist. The erysipelas and diffuse inflammations in general, and epidemic dysentery, may be said to hold an intermediate place between the two great classes of disease now mentioned; the existence of a peculiar morbid poison (whether atmospheric or contagious) in many of these cases being at least highly probable, while the inflammation essential to these diseases is frequently of such intensity as to imply, of itself, great danger. But we think that any one who has duly reflected on the points of distinction above stated, must be convinced that, in every arrangement of acute diseases, the difference of the simply inflammatory from the strictly febrile diseases should be carefully respected; and it need hardly be said, that this has not been done in the present work.

We think it obvious, likewise, that the inflammatory diseases ought to be treated first; because much of the pathology and treatment of the strictly febrile diseases must always turn on the observation of those varieties of local inflammation, which coexist or combine themselves with the more characteristic febrile symptoms, and it is often by observing the deflection from the usual course, and usual effects of inflammations previously explained, that we can best characterize those acute diseases which we regard as of an essentially different class. In this respect, also, the arrangement of our author, although common, appears to us to be injudicious.

Again, under the head of inflammatory diseases, we may easily make an important distinction between those occurring in a constitution previously sound, and producing only the characteristic effects of simple inflammation; and those which occur in a constitution previously disposed to peculiar forms of disease, and modified by that peculiarity of habit. The peculiar results to be expected from inflammation, when occurring, or at least maintaining itself for any considerable time, in one who is already disposed to scrofulous diseases—to gout, probably to rheumatism, to purpura, to asthma, to dropsy, or other constitutional chronic disease, may easily be described, and to a certain extent explained, and distinguished from, although seen to graduate insensibly into those which take place in a perfectly healthy subject, where inflammatory effusion consists of lymph or of pus only, and is followed by gradual absorption of the effused matter. And it is thus that we ought to connect inflammation with these chronic diseases, (whether organic or habitual) which our author merely enumerates as its occasional effects, and afterwards discusses in a totally different part of his work. Reflection on morbid appearances, and especially on the gradual transition of the acknowledged effects of inflammation into other forms of organic disease, will, we apprehend, as clearly illustrate a connexion between inflammation of the serous membranes and the dropsies, between inflammation of the mucous membranes and the profluvia, between inflammation of various healthy textures and the generation of adventitious textures by "perverted nutrition," as it will illustrate the distinction between inflammations and fevers; and no arrangement of the chronic diseases can ever be satisfactory, in which that connexion, and the transition from acute to chronic disease thus effected, is not made one of the principles of classification.

We are aware that, in offering these strictures on the plan and arrangement of the work of Professor Bene, we state objections which apply equally to many other systematic authors, and to our own countrymen quite as much as to continental writers; and we are far from thinking that our knowledge of pathology is so far advanced as to enable us to found on it a complete and strictly natural arrangement of diseases, but we have thought this a good opportunity of stating our conviction, that, in the present state of pathology, all who attempt a systematic arrangement of medical science ought to endeavour to take it as their guide, and strive to make it the basis of a more natural classification of diseases than is yet in use; and that, until this is effected, however much the details of morbid anatomy may be cultivated, the study of pathology, in the wider and more legitimate sense of the term, will not have assumed its due importance, nor produced the beneficial influence which we trust it is yet destined to exert, in facilitating the study and directing the practice of medicine.

